# Genome-wide identification and analysis of mRNA expression in fibroblasts, ES cells, and iPS cells

**DOI:** 10.1016/j.gdata.2015.12.026

**Published:** 2015-12-31

**Authors:** Hiroyuki Hirai, Nobuaki Kikyo

**Affiliations:** a Stem Cell Institute, University of Minnesota, Minneapolis, MN 55455, USA; b Department of Genetics, Cell Biology and Development, University of Minnesota, Minneapolis, MN 55455, USA; c Department of Cardiac Surgery, University of Michigan, Ann Arbor, MI 48109, USA

**Keywords:** Induced pluripotent stem cells, Leukemia inhibitory factor, Oct4, Pluripotency, Reprogramming

## Abstract

Genome-wide expression patterns of mRNA were compared between mouse embryonic fibroblasts (MEFs), embryonic stem cells (ESCs), and various types of induced pluripotent stem cells (iPSCs). iPSCs were established and maintained using modified Oct4 with or without exogenous leukemia inhibitory factor (LIF) and used to identify mRNAs that were potentially involved in the LIF-independence.

The data have been deposited in the NCBI's Gene Expression Omnibus (GEO) database with the accession number GSE65563.

**Table:** 

**Specifications**	
Organism/cell line/tissue	Mus musculus/MEFs, ESCs, and iPSCs
Sex	n/a
Sequencer or array type	Illumina MouseWG-6 v2.0 Beadchips
Data format	Raw and analyzed
Experimental factors	LIF was omitted during establishment and maintenance of M3O-lenti-iPSCs-LIF(−).
Experimental features	Genome-wide expression patterns of mRNAs in MEFs, ESCs, and various iPSCs were compared.
Consent	n/a
Sample source location	n/a

## 1 Direct link to deposited data

http://www.ncbi.nlm.nih.gov/geo/query/acc.cgi?acc=GSE65563

## 2 Experimental design, materials and methods

### 2.1 Cell culture

Cell culture was performed as described [1]. Briefly, MEFs were derived from embryonic day 13.5 of Oct4-GFP transgenic mice (B6;129S4-*Pou5f1tm2Jae*/J; Jackson Laboratory, stock #008214) [2]. MEFs were cultured in fibroblast medium (Dulbecco's Modified Eagle Medium [DMEM] with 10% fetal bovine serum [FBS]). ESCs and iPSCs were cultured in iPSC medium (DMEM, 20% FBS, 100 μM MEM non-essential amino acids, 100 μM 2-mercaptoethanol, and 2 mM l-glutamine, 2 μg/ml doxycycline, and with or without 1000 U/ml LIF depending on the iPSC lines) on irradiated MEF feeder cells. The first 62 amino acids of MyoD were fused to the amino-terminus of the full-length Oct4 to create M3O. Mouse Oct4 and M3O have the FLAG sequence DYKDDDDK at their carboxyl-termini and were cloned into the FUW-tetO doxycycline-inducible lentivirus vector [3]. Oct4 cDNA was also subcloned into the pMXs-IP retroviral vector [4]. Mouse polycistronic Sox2, Klf4, and c-Myc (SKM) were cloned into the pMXs-IP vector [5]. O-retro-iPSCs-LIF (+) were prepared with the combination of retroviral Oct4 and SKM transduced into MEFs and maintained with LIF. O-lenti-iPSCs-LIF (+) were prepared with lentiviral Oct4 and retroviral SKM to maintain the expression of the Oct4 transgene in iPSCs as a control for M3O-based iPSCs. M3O was used instead of Oct4 to prepare M3O-lenti-iPSCs-LIF (+). LIF was omitted during establishment and maintenance to prepare M3O-lenti-iPSCs-LIF (−).

### 2.2 Microarray analysis of mRNA

Gene expression profiling was performed as described [1]. Briefly, total RNA was prepared from MEFs, ESCs (CGR8 and CJ7), and iPSCs with a PureLink RNA Mini Kit (Life Technologies).

RNA was biotinylated and amplified with an Illumina TotalPrep-96 RNA Amplification Kit (Life Technologies). Samples were applied to MouseWG-6 v2.0 Beadchips (Illumina). Hybridization and scanning were performed using the standard Illumina scanning protocol and an iScan Reader (Illumina). Obtained data were analyzed with the Genome Studio (Illumina) and normalized using the rank invariant method.
